# Development and psychometric evaluation of the fear of medical imaging radiation scale (FOMIRS): insights from multimethod analysis

**DOI:** 10.1186/s13244-025-02018-1

**Published:** 2025-06-27

**Authors:** Lin-sen Feng, Si-rong She, Yuan-yuan Zhang, Jia-qi Xie, Zheng-jiao Dong, Ai Tang, Yin-zhu Li, Xiao-qian Wu, Qing Yang, Hao-yu Wang, San-bin Wang

**Affiliations:** 1 The Sixth Affiliated Hospital, Kunming Medical University, Yuxi, China; 2 School of General Practitioners, Kunming Medical University, Yuxi, China; 3 Department of Hematology, The 920th Hospital of Joint Logistics Support Force, Kunming, China; 4 The First School of Clinical Medicine, Kunming Medical University, Kunming, China; 5 School of Basic Medicine, Kunming Medical University, Kunming, China; 6 School of Business, Yuxi Normal University, Yuxi, China; 7 Yiliang County Center for Disease Control and Prevention, Zhaotong, China

**Keywords:** Fear of medical imaging radiation, Scale, Reliability, Validity, Mechanisms

## Abstract

**Objective:**

Fear of medical imaging radiation (FOMIR) may influence disease screening willingness; however, no validated tool currently exists to assess FOMIR. This study aimed to develop and validate the Fear of Medical Imaging Radiation Scale (FOMIRS) and explore its psychological mechanisms.

**Methods:**

Based on classical test theory, the FOMIRS was developed through semi-structured interviews, grounded theory, and Delphi consultation. A cross-sectional survey with 1509 participants was conducted in Yunnan Province from September to December 2024. Psychometric properties were evaluated using construct validity, convergent validity, discriminant validity, criterion-related validity, content validity, and internal consistency. ROC curve analysis was used to determine the critical thresholds. Logistic regression analysis, network analysis, and structural equation modeling were employed to examine the relationships between the FOMIRS and related variables.

**Results:**

The FOMIRS consisted of 18 items organized into a two-dimensional structure. It demonstrated good model fit (Goodness-of-fit index = 0.909, Comparative fit index = 0.949), convergent validity (AVE > 0.45, CR > 0.80), discriminant validity (HTMT = 0.574), criterion-related validity (γ = 0.441), and content validity (S-CVI = 0.889). The FOMIRS also showed excellent internal consistency (Cronbach’s α = 0.926 and McDonald’s ω = 0.935). Cost-induced refusal of imaging examinations, cancer screening willingness, online learning, imaging radiation cognition, and fear of cancer were identified as influencing factors of FOMIR (*p* < 0.05). FOMIR serves as a core node in the network, and imaging radiation cognition may affect cancer screening willingness through this mechanism (*p* < 0.05).

**Conclusion:**

FOMIRS accurately measures individual FOMIR levels. It captures the psychological characteristics and behavioral tendencies associated with FOMIR and indicates potential mechanisms.

**Critical relevance statement:**

We developed the Fear of Medical Imaging Radiation Scale (FOMIRS), a psychometric tool measuring individuals’ fear of medical imaging radiation (FOMIR), demonstrating good reliability, validity, and practical application potential.

**Key Points:**

- Evaluating individuals’ FOMIR improves compliance with imaging exams and reduces related cognitive biases.
- FOMIRS is a reliable and valid tool for measuring FOMIR levels, capturing psychological and behavioral traits, and revealing interactions with external features.
- FOMIR is a complex phenomenon involving psychological traits, behavioral tendencies, and cognitive biases that affect people’s willingness to undergo cancer screening.

**Graphical Abstract:**

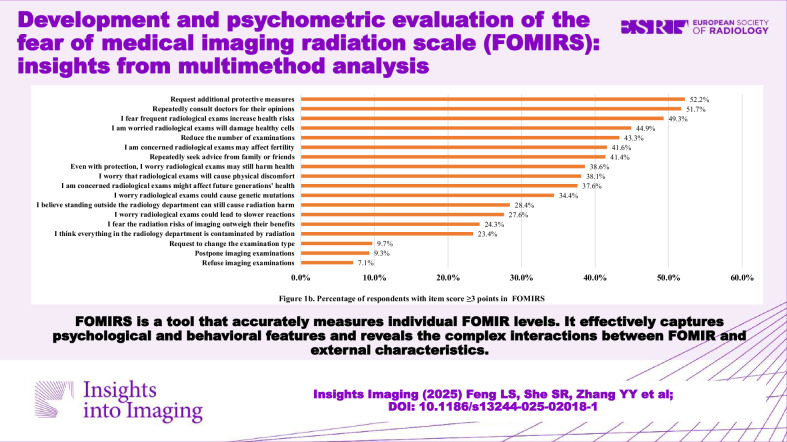

## Background

Excessive exposure to ionizing radiation from CT scans may increase the risk of cancer [1]. In adolescents and children, hematological malignancies and brain cancer may be closely associated with CT radiation exposure [2, 3]. This evidence fully reflects the importance of the appropriate use of CT examinations and radiation dose optimization. However, public misunderstanding and one-sided media coverage of radiation-related cancer risks have resulted in heightened concerns regarding radiation exposure, particularly when dose thresholds and justification of imaging examinations have been overlooked [4].

These misconceptions have contributed to a broader psychological phenomenon known as Fear of Medical Imaging Radiation (FOMIR), a common and subtle negative psychological response among the general population.

Despite professionals’ efforts to correct misconceptions through science education, the public finds it difficult to accurately gauge radiation risks, leading to ongoing cognitive biases [5]. FOMIR may be rooted in the “invisibility” and “uncontrollability” of radiation, with the public’s perception of its harmful effects far exceeding the actual risks—a phenomenon known as the “psychological amplification effect,” which exacerbates fears surrounding various types of radiation. This “psychological amplification effect” further intensifies the public’s fear of different forms of radiation [6].

It is important to note that most medical imaging examinations use ionizing radiation; however, the doses are carefully controlled and maintained within safe limits. Both international and national bodies have developed relevant guidelines and standards [7–9]. Cellular damage caused by low doses of radiation is considered negligible, and reasonable imaging examinations can promote better prognosis with a favorable benefit-to-risk ratio. However, the public may still harbor cognitive biases towards low-dose radiation along with unwarranted anxiety, potentially leading to an imbalance in the risk-benefit assessment of medical decisions [10, 11].

It has been suggested that FOMIR is expressed in X-ray examinations through three common concerns: “All radiation exposure is harmful (carcinogenic),” ”Radiation exposure is cumulative,” and “Children are more susceptible to the effects of radiation” [12]. These beliefs may be particularly pronounced in patients requiring repeated radiological examinations, such as those with periodontal disease or those undergoing dental implantation [13]. Such fear can cause psychological stress among both children and their parents, potentially leading to poor image quality, misdiagnosis, and secondary harm [14].

Additionally, pregnant women are more likely to delay necessary examinations due to concerns about fetal health. In severe gestational diseases such as gestational liver disease, the importance of imaging for the survival of both mother and child is emphasized, with delays potentially leading to fatal consequences [15]. Healthcare structures and providers may also limit the use of X-rays for routine or follow-up evaluations of spinal structure and function owing to irrational pressures caused by FOMIR, thereby further exacerbating the public’s generalized fear of radiation [16–18].

FOMIR has also been implicated as a factor inhibiting willingness to participate in cancer screening. Furthermore, fear of radiation exposure during screening, concerns about discomfort and pain associated with the examination, and anxiety over potential results can all act as deterrents to participation in breast cancer screening [19]. Additionally, the public views radiation exposure and concerns about screening accuracy as drawbacks of lung cancer screening [20].

Integrating insights from existing literature, we conceptualize FOMIR as a complex phenomenon involving psychological traits, cognitive biases, and psychosocial factors. Multiple variables mediated the relationship between FOMIR and related behavioral tendencies. We developed the Fear of Medical Imaging Radiation Scale (FOMIRS) to test this hypothesis. This scale aims to clarify the mechanisms underlying FOMIR by employing multiple methods, thereby providing a scientific basis for understanding FOMIR.

## Materials and methods

### Development of the FOMIRS

The FOMIRS was developed based on classical test theory.

This study, informed by grounded theory, involved semi-structured interviews with 22 participants aged 18–65 in Yunnan Province, China. The interviews, centered on the theme of “Fear of Medical Imaging Radiation,” were conducted according to the “sample saturation principle.”

Questions in semi-structured interviews include:

1. What kind of tests in hospitals do you consider to be medical imaging examinations?
2. Which medical imaging examinations do you think involve radiation exposure?
3. Are you afraid of undergoing medical imaging examinations? If so, why are you afraid of them?
4. What do you think are the adverse effects of radiation exposure during medical imaging examinations?
5. What else do you think about medical imaging examinations and their associated radiation exposure?

The participants included community residents with prior experience in medical imaging as well as imaging physicians and clinicians. Each semi-structured interview lasted between 20 and 45 min and was documented through synchronized note-taking by two trained researchers. Transcripts were created verbatim and annotated to include nonverbal cues such as pauses and gestures. All the records were anonymized and stored in password-protected files for thematic analysis.

NVivo 21 was used to systematically code the raw data, extracting two main constructs: “Fear of Medical Imaging Radiation Psychology (FOMIR-P)” with 11 subthemes, and “Fear of Medical Imaging Radiation Behavior (FOMIR-B)” with seven subthemes. These subthemes guided the development of the Chinese version of the FOMIRS item pool, ensuring that it aligned with Chinese language conventions.

A Delphi expert consultation method was used to refine the initial item pool. Experts from five disciplines (clinical medicine, nursing, public health, applied psychology, and Chinese linguistics) participated in this process. In the first round of consultation, experts reviewed and provided feedback on the content and language of the items. In the second round, each expert rated the content validity of each item on a scale of 0 to 10 with increments of 0.1 points, where higher scores indicated greater relevance to the concept of FOMIR.

Finally, we determined that the Chinese version of the FOMIRS consisted of 18 items. The psychological dimension (FOMIR-P Dimension) contained 11 items scored on a five-point Likert scale. The behavioral dimension (FOMIR-B Dimension) included seven items scored using a three-point Likert scale. All items were positively scored, with higher scores indicating more pronounced FOMIR (Supplementary S1).

### Study tools

Judgmental sampling was employed to select the respondents who met the inclusion and exclusion criteria (Supplementary S2).

A self-assessment electronic questionnaire was developed using *WJX.cn*. The electronic questionnaire consisted of the following sections (Supplementary S3): Home page, Basic information, Radiation awareness, Fear of Cancer Scale (FOCS) [21, 22], and FOMIRS.

### Data collection

The research team conducted a large-sample cross-sectional survey in Yunnan Province from September 7, 2024, to December 31, 2024. Research information, electronic questionnaire links, and digital posters were disseminated to permanent community residents to participate in the study through internal online communication platforms (such as *WeChat* groups, *QQ* groups, and OA office platforms) of local communities or grassroots organizations (excluding medical institutions). Interested participants volunteered to participate in the survey after learning about its purpose and significance. The minimum required sample size was calculated to be *N* = 1067 (Supplementary S4).

### Ethics committee statement and conflict of interest

The study protocol was approved by the Ethics Committee of the Sixth Affiliated Hospital of Kunming Medical University (Approval No. 2024 kmykdx6f014). The research was conducted within the validity period of the ethics approval. Participation in the study was voluntary, anonymous, and noncommercial. Prior to the survey, participants were presented with an electronic informed consent form, and only after indicating their agreement could they complete the questionnaire. Participants could withdraw from the study at any time before submitting the questionnaire, and any entered data could not be saved or uploaded upon withdrawal. The authors declare that they have no conflicts of interest.

### Statistic analysis

Electronic questionnaire data were exported, and a database was constructed using SPSS 26.0. Statistical analyses were performed using SPSS version 26.0, AMOS version 24.0, and Python version 3.11.0.

First, the current status of the respondents’ FOMIR and their perceptions of imaging radiation were analyzed descriptively.

Second, the FOMIRS was tested for the reliability and validity of the FOMIRS. The construct validity of FOMIRS was assessed using both exploratory factor analysis (EFA) and confirmatory factor analysis (CFA). Content validity was evaluated using the Item-level Content Validity Index (I-CVI), Scale-level Content Validity Index (S-CVI), and Spearman correlation analysis. Convergent validity was assessed using the average variance extracted (AVE) and composite reliability (CR) values. Discriminant validity was evaluated by cross-loading comparisons and the heterotrait-monotrait ratio (HTMT). Criterion-related validity was tested using the FOCS as a reference system. Reliability was assessed using Cronbach’s α, McDonald’s ω, and Spearman-Brown coefficients.

Third, the ROC curve of the FOMIRS score was plotted, using whether one feared imaging examinations as the reference variable. This allowed for the determination of a threshold in the measured FOMIRS score to distinguish between high- and low-risk populations for FOMIR. A binary logistic regression model was used to analyze the factors influencing FOMIR.

Additionally, network analysis and structural equation modeling (SEM) were utilized to examine the relationship between variables related to FOMIR and to elaborate on the mechanisms of action underlying this fear, particularly its potential impact on the willingness to undergo cancer screening.

The significance level (α) was set at 0.05.

## Results

### Descriptive analysis

A total of 1666 participants accessed the online questionnaire, with 1509 valid responses (90.58% response rate). The socio-demographic characteristics of the respondents are presented in Supplementary Table 1.

**Fig. 1:**
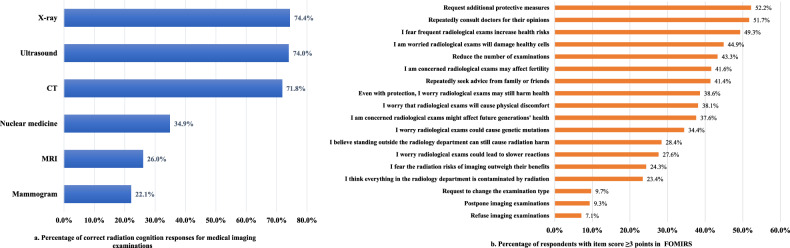
**a** Percentage of correct radiation cognition responses for medical imaging examinations. **b** Percentage of respondents with item score ≥ 3 points in FOMIRS

Awareness of radiation from six common imaging examinations was generally low, and FOMIR was relatively prevalent. Only 504 (33.4%) participants answered correctly with a passing grade (≥ 4/6), and 105 (7.0%) participants achieved a full score (Fig. 1a). FOMIR manifested differently, with an item-level prevalence ranging from 7.1% to 52.2% (item score ≥ 3) (Fig. 1b).

### Construct validity: EFA

The Kaiser-Meyer-Olkin (KMO) measure was 0.937, indicating its high suitability for factor analysis. Bartlett’s test of sphericity yielded a χ² value of 20906.549 (*df* = 153, *p* < 0.001), confirming the appropriateness of the data for EFA.

Principal component analysis with varimax rotation indicated that, although the three-factor model had a higher cumulative variance contribution rate (CVCR) (69.95%), its factors were ambiguous. The two-factor model, with a CVCR of 61.21%, showed a better alignment with the theoretical design of the scale. Consequently, we chose the two-factor model because of its clear interpretation and consistency with the theoretical framework (Table 1).

**Table 1:** Exploratory factor analysis of FOMIRS: a two-factor structure

Item	F1: FOMIR-P	F2: FOMIR-B
FOMIRS 1	**0.575**	0.229
FOMIRS 2	**0.655**	0.225
FOMIRS 3	**0.805**	0.222
FOMIRS 4	**0.827**	0.138
FOMIRS 5	**0.838**	0.163
FOMIRS 6	**0.862**	0.154
FOMIRS 7	**0.880**	0.150
FOMIRS 8	**0.867**	0.184
FOMIRS 9	**0.847**	0.229
FOMIRS 10	**0.786**	0.295
FOMIRS 11	**0.695**	0.353
FOMIRS 12	0.060	**0.815**
FOMIRS 13	0.071	**0.839**
FOMIRS 14	0.094	**0.822**
FOMIRS 15	0.307	**0.464**
FOMIRS 16	0.350	**0.492**
FOMIRS 17	0.321	**0.556**
FOMIRS 18	0.279	**0.609**

The boldface indicates that the item belongs to the corresponding factor. Specifically, FOMIRS 1 to FOMIRS 11 belong to Factor 1 (F1), and FOMIRS 12 to FOMIRS 18 belong to Factor 2 (F2).

### Construct validity: CFA

**Fig. 2:**
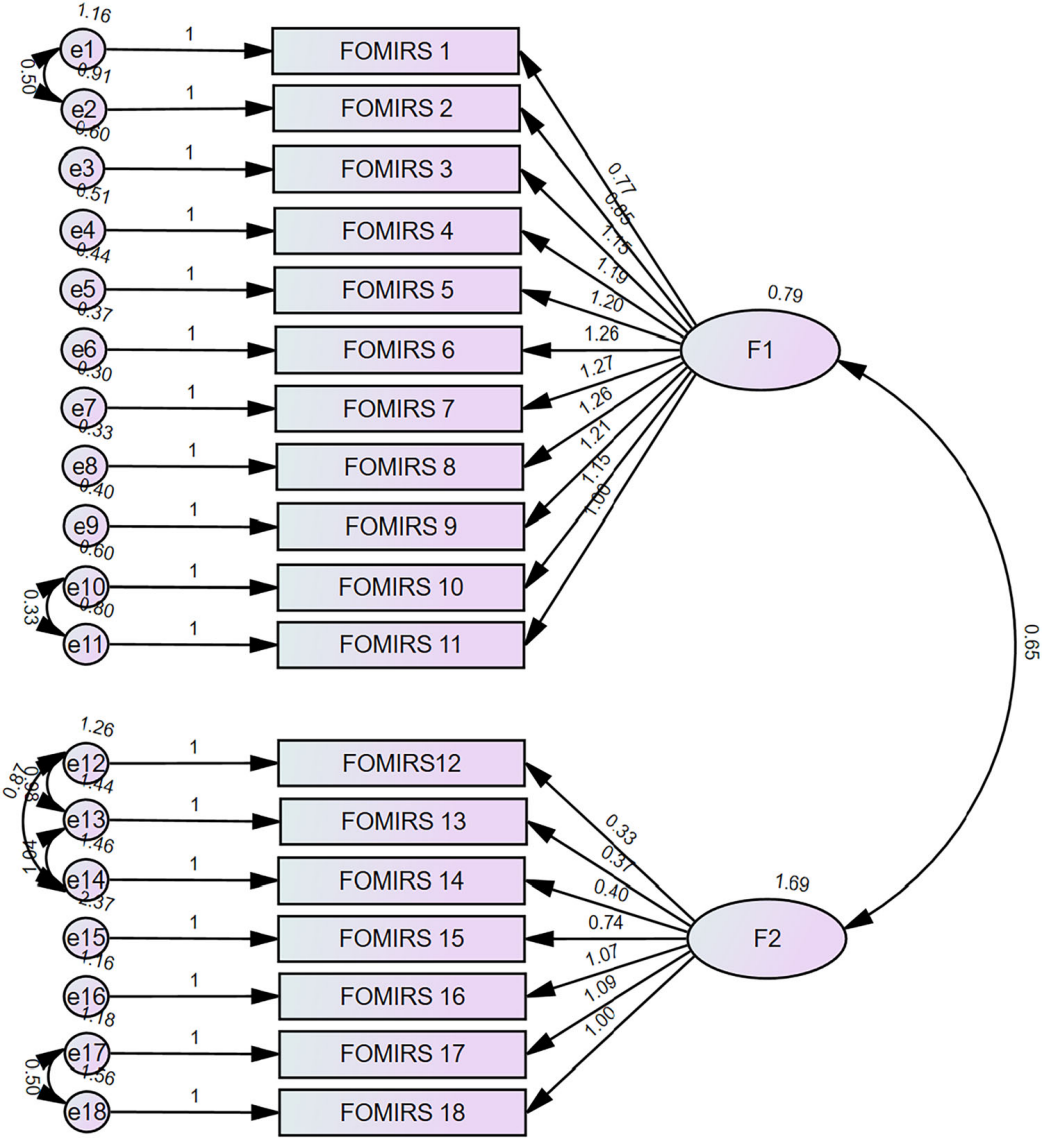
Structural equation model for two-dimensional FOMIRS

However, the uncorrected two-factor model did not achieve the desired fit. However, after correcting for the six residuals, the modified two-factor model exhibited significantly improved fit indices. The parameter estimate between Factor 1 (F1) and Factor 2 (F2) was 0.646 (*p* < 0.001) (Fig. 2, Table 2).

**Table 2:** Goodness-of-fit for structural equation modeling

Model	GFI	AGFI	RMSEA	CFI	NFI	TLI	IFI	RFI
Uncorrected two-factor FOMIRS model	0.717	0.639	0.141	0.807	0.802	0.780	0.807	0.774
Corrected two-factor FOMIRS model	0.909	0.878	0.074	0.949	0.943	0.939	0.949	0.932
Multiple mediation effect model	0.990	0.976	0.049	0.964	0.954	0.930	0.964	0.913
Critical value	> 0.90	> 0.85	< 0.08	> 0.90	> 0.90	> 0.90	> 0.90	> 0.90

*AGFI* Adjusted goodness-of-fit index, *CFI* Comparative fit index, *GFI* Goodness-of-fit index, *IFI* Incremental fit index, *NFI* Normed fit index, *RFI* Relative fit index, *RMSEA* Root mean square error of approximation, *TLI* Tucker-Lewis index

F1 is named “Psychology,” and primarily describes an individual’s FOMIR experience and emotional disturbances. This factor reflects an individual’s tendency to exaggerate, overinterpret, engage in erroneous reasoning, and make subjective assumptions about the negative effects of radiation from imaging examinations.

F2 is named “Behavior,” which primarily describes the behavioral tendencies or action patterns that an individual’s FOMIR may cause. This factor mainly manifests as hesitancy, refusal, avoidance, and active seeking of excessive radiological protection during imaging.

### Convergent validity

F1 showed an AVE of 0.626 and a CR of 0.948, indicating good convergent validity. F2 had an AVE of 0.454 and CR of 0.847, suggesting acceptable convergent validity.

### Discriminant validity

As shown in Table 1, the absolute value of the factor loading for each item in its respective dimension was greater than 0.4, whereas the cross-loadings in other dimensions were less than 0.4.

Furthermore, the HTMT between F1 and F2 was 0.574, which is less than the threshold of 0.85. Collectively, these results indicate that the FOMIRS has good discriminant validity.

### Criterion-related validity

The predeveloped FOCS was the reference standard [18], with a Cronbach’s alpha of 0.948. Spearman’s correlation analysis showed that FOCS was significantly correlated with FOMIRS scores (*rho* = 0.441, *p* < 0.001). Further analysis revealed correlations of *rho* = 0.437 (*p* < 0.001) with the psychological dimension and *rho* = 0.319 (*p* < 0.001) with the behavioral dimension.

### Content validity

Kendall’s concordance coefficient for expert content validity ratings of the 18 items was 0.406 (*p* = 0.007), indicating good scorer reliability.

The I-CVI for the FOMIRS ranged from to 0.852–0.916 (Supplementary Table 2). The S-CVI was calculated using expert ratings ≥ 8 as a high evaluation criterion. Items 11 and 18 each had one expert rating below eight, resulting in an S-CVI of 0.889 (> 0.80).

**Fig. 3:**
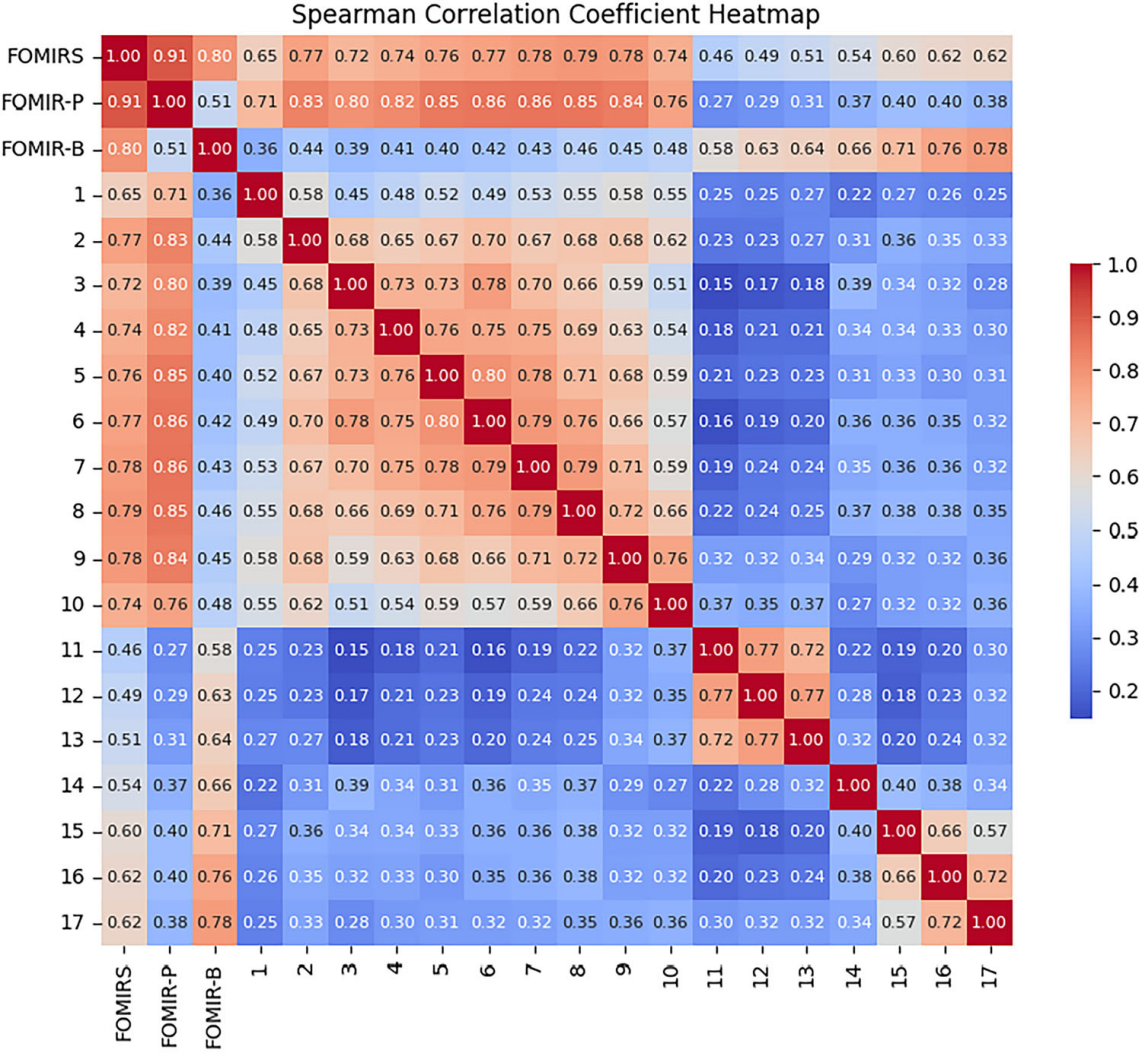
Spearman correlation matrix of FOMIRS, psychological dimension, behavioral dimension and items

Spearman’s correlation analysis (Fig. 3) revealed strong associations between the FOMIRS and its two dimensions (*rho* = 0.91 and 0.80, *p* < 0.05). Item-scale correlations ranged from 0.46 to 0.79 (*p* < 0.05), supporting good content validity.

### Internal consistency

The FOMIRS showed strong internal consistency (Cronbach’s α = 0.926, McDonald’s ω = 0.935) with good values for F1 (α = 0.950, ω = 0.951) and F2 (α = 0.823, ω = 0.833). The Spearman-Brown coefficient for odd-even split-half reliability was 0.960 overall, 0.965 for F1, and 0.887 for F2, indicating excellent reliability across the scale.

### Measurement threshold of FOMIRS

“Whether fear of imaging examination” was used as the target variable to plot the ROC curve for the FOMIRS score (Supplementary Fig. 1). The area under the curve was 0.876 (95% CI: 0.858–0.893, *p* < 0.001).

The Yoden Index reached a maximum value of 0.594 at a FOMIRS score threshold of 38.5, with 76.6% sensitivity and 82.8% specificity. This suggests that 38.5 is the critical threshold between high fear risk (FOMIRS score ≥ 39) and low fear risk (FOMIRS score ≤ 38) (Supplementary Table 3).

### FOMIR’s influencing factors

In this study, 959 (63.6%) respondents were categorized as having low fear risk and 550 (36.4%) as having high fear risk. The binary Logistic Regression Model (Table 3) showed that cost-induced refusal of imaging examinations, cancer screening willingness, online learning of medical imaging knowledge (online learning), imaging radiation cognition, and fear of cancer were identified as factors influencing FOMIR (*p* < 0.05). Age, sex, ethnicity, education, and place of residence were not factors influencing FOMIR.

**Table 3:** Binary logistic regression analysis of factors influencing FOMIR

Variable	Subgroup	*β*	S.E.	*p*-value	95% CI
Cost-induced refusal of imaging examinations (Reference variable: Rejection)					
	No rejection	−1.363	0.263	< 0.001	0.153–0.429
	Uncertainty	−0.754	0.259	0.004	0.283–0.782
Cancer screening willingness (Reference variable: Willingness)					
	Unwillingness	0.604	0.186	0.001	1.271–2.632
	Uncertainty	−0.039	0.148	0.790	0.720–1.284
Online learning (Reference variable: Yes)					
	No	0.344	0.146	0.019	1.059–1.880
Imaging radiation cognition (Reference variable: Passing)					
	Failing	−0.257	0.123	0.036	0.608–0.984
Fear of cancer		0.045	0.004	< 0.001	1.038–1.054

Dependent variable:

FOMIR: 1 = low fear risk, 2 = high fear risk

Independent variable:

Cost-induced refusal of imaging examinations: 1 = No rejection, 2 = Uncertainty, 3 = Rejection

Cancer screening willingness: 1 = Unwillingness, 2 = Uncertainty, 3 = Willingness

Online learning: 1 = No, 2 = Yes

Imaging radiation cognition: 1 = Failing (< 4/6), 2 = Passing (≥ 4/6)

Fear of cancer (FOCS score): 0–68 point

### Network analysis of FOMIR’s external structures

**Fig. 4:**
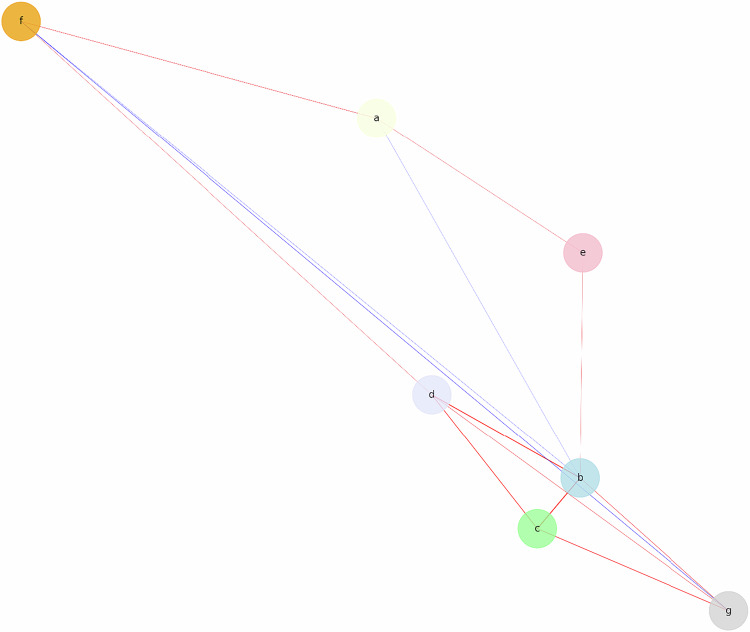
Network analysis topological graph of the FOMIR external structures

An undirected weighted network, G = (V, E), was constructed to identify significant correlations (|*rho*| ≥ 0.05) based on the Spearman correlation coefficient matrix of the seven core variables (nodes a-g). The network density is 0.619, and the centrality indices are listed in Table 4. In the topological graph, the red edges indicate a positive correlation, whereas the blue edges indicate a negative correlation (Fig. 4).

**Table 4:** Centrality measures in the external structure network analysis of FOMIR

Node	Meaning	Betweenness	Closeness	Strength
a	Online learning	0.0667	0.0602	0.1416
b	FOMIR-P	0.2000	0.0932	0.7025
c	FOMIR-B	< 0.0001	0.0917	0.7529
d	Fear of cancer	< 0.0001	0.0861	0.7049
e	Imaging radiation cognition	< 0.0001	0.0547	0.1432
f	Cancer screening willingness	0.2000	0.0766	−0.0005
g	Cost-induced refusal of imaging examinations	0.1333	0.0860	0.3091

Node b exhibited the highest betweenness centrality (0.2000) and closeness centrality (0.0932) along with a relatively high strength (0.7025), establishing it as a pivotal node and core bridge in the network.

Node f also exhibited the highest betweenness centrality (0.2000) but with the lowest negative strength (−0.0005) and relatively low closeness centrality (0.0766), suggesting that node f may influence the network through negatively correlated long paths, potentially involving other mediating effects.

Nodes c and d had high strengths (0.7529 and 0.7049, respectively), indicating strong connections to the core nodes and a significant influence on the network. Node g showed relatively high betweenness centrality (0.1333), highlighting its role as a mediator. The other nodes were primarily peripheral nodes that played a supplementary role in the network.

### SEM of the FOMIR mechanisms

**Fig. 5:**
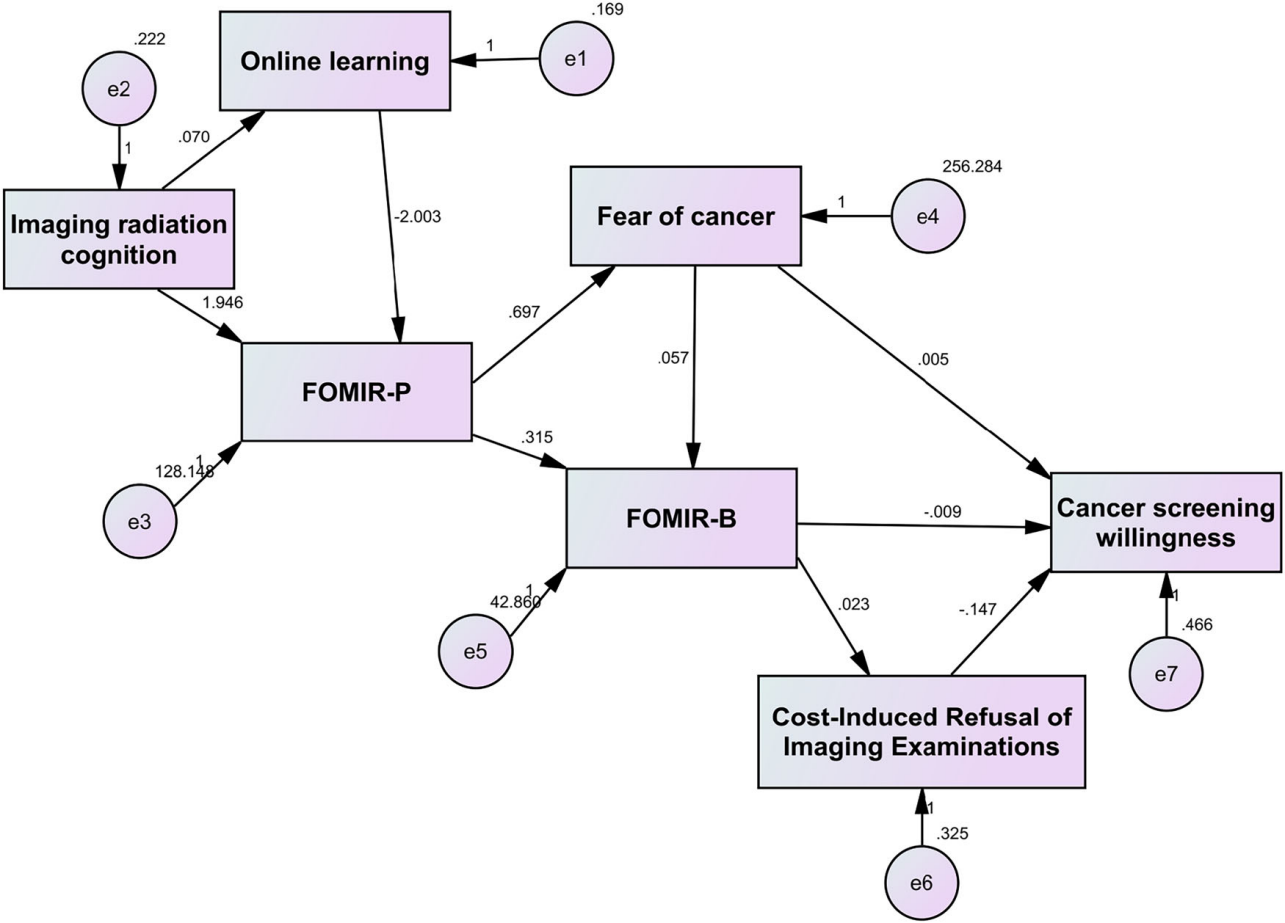
Structural equation modeling of the mechanisms underlying FOMIR

Based on these hypotheses, SEM was used to explore the mechanisms of the seven core variables. The best-fitting SEM (Table 5, Fig. 5), with online learning and cost-induced refusal of imaging examinations as mediators, had the lowest Akaike Information Criterion (84.834) and Bayesian Information Criterion (175.260) values, and demonstrated an optimal fit (Table 2).

**Table 5:** Parameter estimation of a multiple mediation model for FOMIR

Independent variable			Dependent variable			
	Online learning	FOMIR-P	Fear of cancer	FOMIR-B	Cost-induced refusal of imaging examinations	Cancer screening willingness
Imaging radiation cognition	0.070*	1.946*	—	—	—	—
Online learning	—	−2.003*	—	—	—	—
FOMIR-P	—	—	0.697**	0.315**	—	—
Fear of cancer	—	—	—	0.057**	—	0.005**
FOMIR-B	—	—	—	—	0.023**	−0.009**
Cost-induced refusal of imaging examinations	—	—	—	—	—	−0.147**

* p < 0.01, ** *p* < 0.001

The model contained two key paths:

1. Core Path: Imaging radiation cognition → FOMIR-P → FOMIR-B → cancer screening willingness [*β* = −0.0055].
2. Competing Path: Imaging Radiation Cognition → FOMIR-P → fear of cancer → cancer screening willingness [*β* = 0.0068].

## Discussion

### Dimensional deconstruction of FOMIR: dual drive of psychology and behavior

This study proposed a theoretical framework for the FOMIR and developed an 18-item FOMIRS. Designed to measure individual fear of FOMIR, the scale has a two-dimensional structure. Empirical studies with large sample sizes have confirmed the validity of these methods. The results showed that the FOMIRS has good structural, content, convergent, discriminant, criterion-related validity and excellent internal reliability.

The psychological dimension focuses on excessive concerns about disease risk and genetic variation, cognitive distortions such as exaggerated perceptions of harm, and emotional responses such as anxiety and worry related to imaging radiation. This reflects “risk perception amplification,” where the public overestimates low-dose radiation risks, ignores dose thresholds and individual differences, and underestimates clinical benefits. Nearly half of the respondents feared potential impacts on core life functions such as reproduction and genetics.

The behavioral dimension focuses on behavioral tendencies due to fear, reflecting the outward manifestation of psychological concerns. High-frequency (≥ 40%) fear behaviors included overprotection, repeated consultations, hesitation, and direct intervention in medical processes, potentially increasing medical costs and time. In contrast, low-frequency (≤ 30%) fear behaviors involve avoiding examinations and requesting changes to procedures, which may delay diagnosis and treatment.

Notably, although most imaging examinations do not require extra protection, 52.2% of behaviors included requests for additional protective measures, showing a public reliance on these measures to reduce anxiety. Frequently consulting others (doctors, friends, and relatives) and requesting protection indicate mistrust in doctor-patient communication. Individuals may feel that doctors have not fully explained the necessity and risks of the examination. They seek repeated reassurance to decrease anxiety, fear, and uncertainty and sometimes ask for changes in clinical decisions to bridge trust gaps.

Additionally, individuals perceived high examination costs were positively associated with FOMIR behavior. This indicates that, when patients refuse examinations because of cost concerns, their decision-making logic prioritizes economic factors over risk considerations. This reflects a distrust in medical advice, suggesting that they may believe that “examinations are unnecessary and doctors prescribe them primarily for financial gain.” Fortunately, despite the prevalence of fear, the rate of complete refusal to undergo testing is low, suggesting that when the public repeatedly weighs fear against health needs, they may be more inclined to undergo examination but seek psychological compensation through “protective” and “counseling.”

### The potential impact of public cognitive bias on medical imaging radiation

This study revealed a widespread cognitive bias in the public’s perception of medical imaging radiation, highlighting the confusion about the attributes of different imaging modalities. MRI and ultrasound do not rely on ionizing radiation. However, 74.0% and 26.0% of respondents mistakenly believed that MRI and ultrasound involve radiation, respectively. This suggests that the public may generally perceive imaging exams as “harmful” and “radiation-associated.”

In clinical practice, individuals who misinterpret “radiation” exams as “non-radiation” may underestimate the actual risks, while those who incorrectly view “non-radiation” exams as “radiation” may avoid necessary tests. This leads to a “cognitive polarization” phenomenon, where misunderstandings about the nature of imaging examinations can result in unnecessary fear or underestimation of risks.

Among the traditional assumptions, it is generally believed that correct cognition reduces psychological distress [23]. However, this study found that accurate imaging radiation cognition may promote FOMIR psychology, while learning-related knowledge through the Internet has a weaker buffering and inhibitory effect on this promotion. There are two potential reasons for this observation:

1. Heightened Risk Awareness: Correct cognition may lead individuals to more precisely recognize the potential risks of radiation. This clear risk perception can exacerbate fear and lead to anxiety.
2. Superficial Understanding: Correct cognition might remain superficial, lacking an understanding of imaging technology principles and the risk-benefit ratio of radiation. This can lead to an overestimation of the radiation risk and trigger more intense anxiety.

The study found that Internet-based knowledge can significantly reduce fear of imaging radiation, as indicated by a large coefficient value. This finding suggests that online information is crucial for alleviating fear, especially when correcting misconceptions. However, it is important to recognize that online platforms can provide accurate scientific information and spread exaggerated risk information. Exposure to authoritative science communication helps individuals understand the real radiation risks and reduce their fear.

### The complex mechanisms underlying FOMIR

When an individual experiences FOMIR, their response depends on whether their FOMIR-related behavioral tendency (FOMIR-B) is dominant, or whether the fear of the disease (e.g., cancer) itself is dominant.

If an individual is more concerned about the potential harm from radiation, fear can directly influence their behavior through emotional spillover effects, reducing their willingness to undergo disease screening. In this scenario, the financial burden may become a key factor affecting screening decisions. Financial pressure can be a significant barrier even if an individual is willing to participate in disease screening. However, the effect of the financial burden is relatively weak.

However, if fear of the disease is dominant, it may promote awareness of “early screening and treatment,” increasing individuals’ willingness to undergo screening, and improving screening compliance. However, this can have both positive and negative effects. Moderate fear can enhance health literacy and encourage screening, but excessive fear can lead to anxiety and worsen FOMIR-related behavioral tendencies (FOMIR-B), ultimately reducing willingness to be screened.

### Limitations

Because of the study’s anonymous design, the test-retest reliability of the FOMIRS could not be assessed. This limits our ability to fully evaluate the temporal stability and consistency of the scale across repeated measurements. However, the strong internal consistency and split-half reliability of the scale suggests good stability over time.

As this study employed non-random sampling and recruited participants through community-based online platforms, there may be a potential sample selection bias; for example, it could be difficult to reach residents who use mobile phones or online platforms less frequently. In future, we will further investigate the current status of FOMIR in different populations.

Given the cross-sectional nature of the data, this study can provide insights into FOMIR mechanisms but cannot establish causal relationships. Although the model identified statistically significant associations and potential pathways, it was limited in its ability to confirm temporal or causal directions owing to the lack of dynamic data and a limited set of variables. However, longitudinal studies are required to address these limitations. Considering small β values, the actual impact should be interpreted in the context of other factors. Further research is needed to address this issue.

## Conclusion

The FOMIRS is a tool that accurately measures individual FOMIR levels. This provides strong support for an in-depth understanding of public attitudes toward medical imaging examinations and ionizing radiation, thus addressing the gap in quantitative FOMIR research.

## Supplementary information

ELECTRONIC SUPPLEMENTARY MATERIAL

## Data Availability

Researchers may use FOMIRS free of charge for their own research purposes, provided they obtain the corresponding author’s permission and no commercial interests are involved. The data used and analyzed during the current study are available from the corresponding author upon reasonable request.

## Abbreviations

AVE: Average variance extracted
CFA: Confirmatory factor analysis
CR: Composite reliability
CVCR: Cumulative variance contribution rate
EFA: Exploratory factor analysis
F1: Factor 1
F2: Factor 2
FOCS: Fear of cancer scale
FOMIR: Fear of medical imaging radiation
FOMIR-B: Fear of medical imaging radiation-behavior
FOMIR-P: Fear of medical imaging radiation-psychology
FOMIRS: Fear of medical imaging radiation scale
HTMT: Heterotrait-monotrait ratio
I-CVI: Item-level content validity index
S-CVI: Scale-level content validity index
SEM: Structural equation model
