# Supplementary material for: Development and psychometric evaluation of the fear of medical imaging radiation scale (FOMIRS): insights from multimethod analysis
Source: Insights Imaging. 2025 Jun 27;16:140. doi: 10.1186/s13244-025-02018-1 (PMC12205105; doi:10.1186/s13244-025-02018-1)
Supplement: Supplementary file 1 — ELECTRONIC SUPPLEMENTARY MATERIAL [file 13244_2025_2018_MOESM1_ESM.pdf]

Development and Psychometric Evaluation of the Fear of Medical Imaging Radiation Scale (FOMIRS): Insights from Multimethod Analysis

ELECTRONIC SUPPLEMENTARY MATERIAL

Insights Imaging (2025) Feng LS, She SR, Zhang YY, et al.

S1. Fear of Medical Imaging Radiation Scale (FOMIRS) and Scoring method

Item No.	Item	scoring method				
**Do you agree with the following statements and thoughts regarding medical imaging examinations?**		**Strongly Agree**	**Somewhat Agree**	**Unsure**	**Somewhat Disagree**	**Strongly Disagree**
**FOMIRS 1**	I believe standing outside the radiology department can still cause radiation harm.	4	3	2	1	0
**FOMIRS 2**	I think everything in the radiology department is contaminated by radiation.	4	3	2	1	0
**FOMIRS 3**	I worry that radiological exams will cause physical discomfort.	4	3	2	1	0
**FOMIRS 4**	I fear frequent radiological exams increase health risks.	4	3	2	1	0
**FOMIRS 5**	I am concerned radiological exams may affect fertility.	4	3	2	1	0
**FOMIRS 6**	I worry radiological exams could cause genetic mutations.	4	3	2	1	0
**FOMIRS 7**	I am worried radiological exams will damage healthy cells.	4	3	2	1	0
**FOMIRS 8**	I am concerned radiological exams might affect future generations' health.	4	3	2	1	0
**FOMIRS 9**	Even with protection, I worry radiological exams may still harm health.	4	3	2	1	0
**FOMIRS 10**	I worry radiological exams could lead to slower reactions.	4	3	2	1	0
**FOMIRS 11**	I fear the radiation risks of imaging outweigh their benefits.	4	3	2	1	0

Would you experience any of the following situations due to fear or concern about the radiation risks from imaging examinations?		Yes	Not sure	No
**FOMIRS 12**	Refuse imaging examinations	4	2	0
**FOMIRS 13**	Postpone imaging examinations	4	2	0
**FOMIRS 14**	Request to change the examination type	4	2	0
**FOMIRS 15**	Reduce the number of examinations	4	2	0
**FOMIRS 16**	Request additional protective measures	4	2	0
**FOMIRS 17**	Repeatedly consult doctors for their opinions	4	2	0
**FOMIRS 18**	Repeatedly seek advice from family or friends	4	2	0

S2. Inclusion and Exclusion Criteria

Judgmental sampling was employed to select respondents meeting the following inclusion criteria and exclusion criteria:

(1) Voluntary and informed consent to participate in the study;

(2) Adults aged 18 years or older;

(3) Non-healthcare workers (including doctors, nurses, medical technicians, and pharmacy technicians);

(4) No past or current history of cancer or malignancy;

(5) Ability to operate, read, and comprehend the electronic questionnaire.

Exclusion criteria included participants who failed the honesty item test.

S3. Composition of the Electronic Questionnaire

The electronic questionnaire comprised the following sections:

(1) **Home page:** Including the title of the questionnaire, informed consent; screening items for minors, healthcare workers, and individuals with a history of cancer or malignancy;

(2) **Basic information:** Covered demographic details such as age, gender, ethnicity, place of residence, and educational level. Also included questions about fear of imaging examinations, whether they acquire imaging examination knowledge via the internet (hereinafter referred to as online learning), the likelihood of cost-induced Refusal of Imaging Examinations, and cancer screening willingness.

(3) **Radiation awareness:** Respondents were asked to judge whether radiation is involved in six types of imaging examinations: X-ray radiography, ultrasound, CT, MRI, nuclear medicine examination, and mammography.

(4) **Fear of Cancer Scale (FOCS):** This scale was used to evaluate the level of fear of cancer among non-cancer patients. Higher scores indicated a more pronounced fear of cancer among respondents. The scale exhibits good cross-cultural adaptability

(5) **Chinese version of FOMIRS.**

S4. Sample Size Estimation

Since the total population of this study could not be estimated, the sample size was calculated using the formula.

$$N = \frac{t^2 \times p(1-p)}{e^2}$$

With p set to 0.5, a confidence level of 95% was specified, corresponding to a critical value of t=1.96 and an allowable sampling error of e=3%, and the minimum sample size was calculated to be N=1067.

Supplementary Table 1. Univariate analysis of respondents' general characteristics and FOMIR

Variable	Grouping	N（%）		Percentage of high fear risk （%）	*P*
**Age**					0.265
	18-25 years	286	(19.0%)	31.8%	
	26-35 years	389	(25.8%)	39.1%	
	36-45 years	625	(41.4%)	36.5%	
	≥46 years	209	(13.9%)	37.8%	
**Gender**					0.584
	male	538	(35.7%)	37.4%	
	female	971	(64.3%)	35.9%	
**Nationality**					0.780
	Han	1160	(76.9%)	36.6%	
	Other minorities	349	(23.1%)	35.8%	
**Education background**					0.797
	Junior high school and below	366	(24.3%)	35.8%	
	Highschool/vocational /technical secondary school	354	(23.5%)	38.1%	
	Junior college	244	(16.2%)	37.7%	
	Bachelor degree or above	545	(36.1%)	35.2%	

**Residence**				0.044
Urban area	1057	(70.0%)	34.8%	
non-urban area	452	(30.0%)	40.3%	
**Cost-Induced Refusal of Imaging Examinations**				＜0.001
Rejected	88	(5.8%)	60.2%	
Not sure	724	(48.0%)	43.1%	
Not reject	697	(46.2%)	26.5%	
**Cancer screening willingness**				0.005
Willingness	1020	(67.6%)	35.2%	
Uncertainty	310	(20.5%)	34.2%	
Unwillingness	179	(11.9%)	47.5%	
**Online learning**				0.016
Yes	328	(21.7%)	30.8%	
No	1181	(78.3%)	38.0%	
**Imaging radiation cognition**				0.021
Pass（≥4/6）	504	(33.4%)	40.5%	
Failing（≤3/6）	1005	(66.6%)	34.4%	

Supplementary Table 2. Item-level Content Validity Index（I-CVI）

Item	I-CVI	Item	I-CVI
**Item 1**	0.912	**Item 10**	0.902
**Item 2**	0.904	**Item 11**	0.852
**Item 3**	0.866	**Item 12**	0.868
**Item 4**	0.896	**Item 13**	0.876
**Item 5**	0.916	**Item 14**	0.904
**Item 6**	0.874	**Item 15**	0.882
**Item 7**	0.864	**Item 16**	0.858
**Item 8**	0.908	**Item 17**	0.886
**Item 9**	0.910	**Item 18**	0.854

Supplementary Table 3. Sensitivity, specificity, and Yoden index for each FOMIRS score threshold

Threshold	Sensitivity	Specificity	Yoden index
28.5	94.7%	53.8%	0.484
29.5	94.1%	55.7%	0.498
30.5	93.9%	57.6%	0.514
31.5	92.8%	59.5%	0.524
32.5	91.2%	61.1%	0.523
33.5	88.1%	64.0%	0.521
34.5	85.7%	66.7%	0.524
35.5	83.4%	69.2%	0.526
36.5	81.4%	76.1%	0.575
37.5	78.9%	79.2%	0.581
**38.5**	**76.6%**	**82.8%**	**0.594**
39.5	73.4%	84.4%	0.578
40.5	71.5%	86.5%	0.580
41.5	67.6%	87.3%	0.549
42.5	64.8%	88.8%	0.536
43.5	62.3%	91.0%	0.533
44.5	58.0%	92.4%	0.504
45.5	53.9%	93.5%	0.474
46.5	51.2%	94.3%	0.455
47.5	47.1%	95.0%	0.421
48.5	44.1%	96.1%	0.401

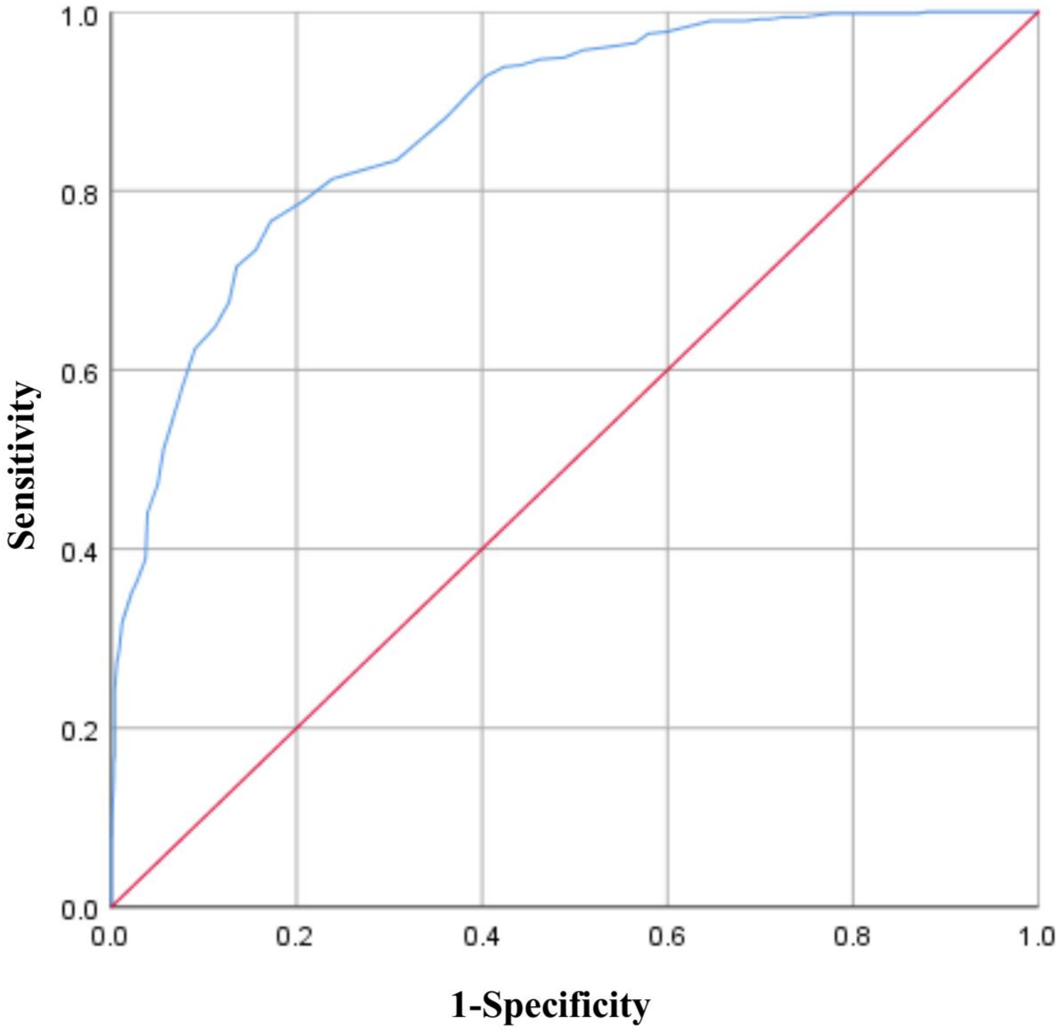

Supplementary Figure 1. ROC curve analysis of FOMIRS scores
